# Manufacturing Process of Hyaluronic Acid Dermal Fillers

**DOI:** 10.3390/polym16192739

**Published:** 2024-09-27

**Authors:** Gi-Woong Hong, Jovian Wan, Youngjin Park, Jane Yoo, Hugues Cartier, Sebastien Garson, Diala Haykal, Kyu-Ho Yi

**Affiliations:** 1 Samskin Plastic Surgery Clinic, Seoul 06577, Republic of Korea; cosmetic21@hanmail.net; 2 Asia Pacific Aesthetic Academy, Hong Kong; jovian.wan@apaa.org; 3 Obliv Clinic, Incheon 21998, Republic of Korea; youngjinp@gmail.com; 4 Department of Dermatology, Mount Sinai School of Medicine, New York, NY 10029, USA; jane.yoo.md@gmail.com; 5 Centre Médical Saint Jean, 62000 Arras, France; h.cartier@imcas.com; 6 Cabinet Médical, 60300 Senlis, France; s.garson@imcas.com; 7 Centre Laser Palaiseau, 91120 Palaiseau, France; docteur.haykal@gmail.com; 8 BK21 FOUR Project, Division in Anatomy and Developmental Biology, Department of Oral Biology, Human Identification Research Institute, Yonsei University College of Dentistry, 50-1 Yonsei-ro, Seodaemun-gu, Seoul 03722, Republic of Korea; 9 Maylin Clinic (Apgujeong), Seoul 06005, Republic of Korea

**Keywords:** hyaluronic acid fillers, cross-linking, biphasic fillers, monophasic fillers, non-hyaluronic acid fillers, dermal filler

## Abstract

Hyaluronic acid (HA) fillers are extensively utilized in aesthetic medicine due to their biocompatibility, reversibility, and effectiveness in enhancing skin hydration, volume, and overall appearance. These fillers are predominantly produced through microbial fermentation, followed by a critical cross-linking process that enhances their longevity by resisting enzymatic degradation. This review provides a thorough examination of the manufacturing processes that differentiate HA fillers, with particular attention to the distinctions between biphasic and monophasic variants. Unlike previous studies, this review emphasizes the specific cross-linking techniques and their substantial impact on the fillers’ rheological properties, such as elasticity and cohesiveness, which are crucial to their clinical performance and patient outcomes. Additionally, the review offers a comprehensive comparison of HA fillers with non-HA alternatives, including calcium hydroxylapatite, poly-l-lactic acid, and polymethyl methacrylate, highlighting the unique advantages and potential complications associated with each type. By presenting novel insights into the latest advancements and challenges in filler technology, this review aims to provide clinicians with a deeper understanding of filler properties, thereby guiding them in making informed decisions to optimize patient safety and aesthetic results.

## 1. Introduction

In contemporary clinical practice, dermal fillers are primarily classified based on their base materials, with hyaluronic acid (HA) fillers being the most commonly used. This has led to a clear distinction between HA-based fillers and those derived from alternative substances [1]. Currently available non-HA fillers include calcium hydroxylapatite (CaHA), polymethyl methacrylate (PMMA), poly-l-lactic acid (PLLA), collagen, polyacrylamide gel (PAAG), and polycaprolactone (PCL) fillers [2,3,4,5,6,7]. A fundamental difference between these categories is that HA fillers can be enzymatically dissolved if adverse reactions or patient dissatisfaction occur, while non-HA fillers lack such an enzymatic agent for degradation and removal [8,9,10].

As a result, non-HA fillers are considered non-dissolvable, requiring more invasive removal procedures such as dilution, aspiration, or manual expression. In cases where fillers become calcified or hardened, they may only be fragmented through saline injection and pressure application, with the expectation that the body’s phagocytic processes will eventually clear them [11]. However, severe foreign body reactions and inflammation may necessitate surgical excision of the filler, potentially leading to further complications [12,13]. The ability to safely dissolve and remove HA fillers is, therefore, a significant factor contributing to their widespread use and trust among clinicians and patients [8,9,10].

This review provides a comprehensive analysis of the manufacturing processes and material properties of HA dermal fillers, with a particular emphasis on the various cross-linking methods and their implications for clinical outcomes. Unlike previous reviews, which often generalize the differences between biphasic and monophasic fillers, this study delves into the specific manufacturing techniques, such as physical versus chemical cross-linking, and examines how these processes influence the rheological properties and clinical performance of the fillers. Additionally, this review integrates recent advancements in microbial fermentation and purification techniques for HA production, underscoring their impacts on the safety and efficacy of HA fillers. By offering a detailed comparison with non-HA fillers, this work provides novel insights into the advantages and limitations of different filler types, ultimately guiding clinicians in selecting the most appropriate fillers for specific aesthetic applications.

## 2. Hyaluronic Acid Filler

**Figure 1:**
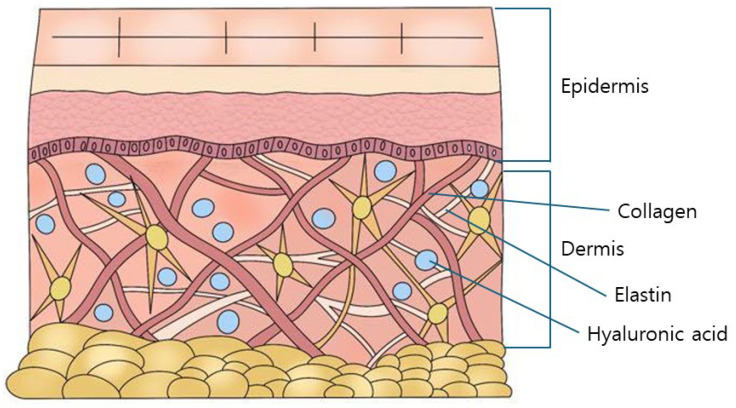
Representation of collagen, elastin, and hyaluronic acid within the skin structure (not to scale).

The skin, although subject to individual variation, is the largest organ of the human body, accounting for approximately 6–7% of total body weight, with an average mass of 3–4 kg [14,15]. Structurally, the skin comprises three distinct layers: the epidermis, the dermis, and the subcutaneous layer. The dermis is particularly significant as it contains key components of the extracellular matrix, including collagen, elastin, and HA (Figure 1) [16,17]. It is estimated that the total HA content in an adult human body ranges from 12 to 15 g, with approximately half of this amount located within the skin [18,19].

HA plays a crucial role in maintaining skin hydration and volume due to its remarkable ability to attract and retain up to 1000 times its molecular weight in water. This capacity for moisture retention is vital for preserving the skin’s softness and elasticity [20]. Additionally, HA facilitates the proliferation and migration of fibroblasts, which are essential for the production of collagen and elastin [20]. Furthermore, HA contributes to the neutralization of reactive oxygen species, thereby mitigating premature aging of skin cells [21,22].

As the body ages, the concentration of HA in the skin gradually diminishes. Up until approximately the age of 20, HA levels remain relatively stable; however, beyond this point, they begin to decline. By the age of 55, the HA content in the skin is typically about half of what it was at age 35. Injecting HA into the dermis of aging skin can lead to increased water absorption, thereby enhancing skin volume and exerting compressive force on surrounding fibroblasts. This compression stimulates fibroblasts to produce new collagen, which subsequently increases the volume of the dermis and subcutaneous tissue [23,24,25,26].

**Figure 2:**
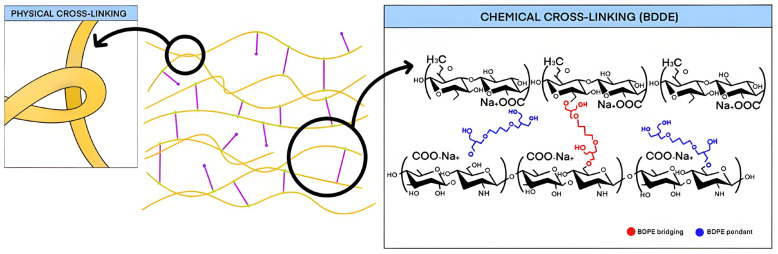
A comparison of non-cross-linked and cross-linked hyaluronic acid (not to scale). This schematic illustrates the differences between natural hyaluronic acid, cross-linked hyaluronic acid, and the process of physical cross-linking. Natural hyaluronic acid is represented by the yellow line, signifying a linear molecule composed of repeating disaccharide units. Cross-linked hyaluronic acid is depicted at a higher magnification, where the yellow line is interconnected with pink links, showing additional bonds formed by 1,4-butanediol diglycidyl ether or 1,2-butanediol diglycidyl ether between the chains. These chemical bonds create a stable network that enhances the longevity of hyaluronic acid fillers in cosmetic applications. The degree of cross-linking varies, influencing the gel’s cohesiveness and resistance to breakdown. Physical cross-linking describes a method of modifying hyaluronic acid without chemical agents, utilizing changes in temperature, pH, or external forces to induce cross-linking.

Naturally occurring HA in the body is degraded by the enzyme hyaluronidase. To effectively utilize HA as a dermal filler, it must undergo a stabilization process known as cross-linking. This process involves chemically or physically bonding HA molecules to prevent their rapid enzymatic degradation, resulting in a more durable form of HA that resists natural breakdown within the body (Figure 2) [27].

HA fillers are designed to enhance the physical and rheological properties of natural HA to prevent its rapid degradation. Given their use in human tissues, these fillers must also exhibit biocompatibility to minimize the risk of adverse tissue reactions [27]. Historically, the raw HA used in these fillers was sourced from animal tissues, such as bovine tongues, cartilage, and chicken combs. However, due to concerns over allergic reactions and the potential for disease transmission, contemporary production predominantly utilizes HA derived through microbial fermentation processes [28,29].

The primary microorganisms employed in HA production via fermentation are strains of *Streptococcus*, particularly *Streptococcus zooepidemicus* [30]. The first HA filler, Restylane, introduced the non-animal stabilized hyaluronic acid (NASHA) process, emphasizing the use of HA that is not derived from animal sources. While the source of HA was initially a critical factor in filler development, the shift to non-animal-derived raw materials has now become the industry standard [31].

HA produced through microbial fermentation is preferred for its high purity and reduced risk of pathogen contamination, a common concern associated with animal-derived materials. However, microbial sources can introduce significant impurities, such as endotoxins, cellular organelles, and cell-derived proteins, necessitating extensive purification processes [32]. Typically, HA is dissolved in a sodium hydroxide (NaOH) solution and undergoes a series of purification steps, including centrifugation, filtration, precipitation, and adsorption, to produce the raw HA used in fillers. The complexity of these purification processes results in varying levels of HA purity, which are categorized into food grade, cosmetic grade, and medical grade [33].

Medical-grade HA, which undergoes the most rigorous purification, is utilized in various applications, including intraocular lens fillers, intra-articular injections, adhesion prevention, and scaffolds for tissue engineering, in addition to being the primary ingredient in HA fillers. This grade of HA, specifically intended for injection, is significantly more expensive than HA used for ophthalmic applications and is considered the purest form available [34].

### 2.1. Classification of Hyaluronic Acid Fillers According to Their Production Processes

The classification of HA fillers is commonly divided into biphasic and monophasic variants, although these terms lack strict scientific definition. Biphasic fillers are characterized by their palpable granular consistency, which imparts a gritty sensation upon handling, whereas monophasic fillers are distinguished by a smoother, uniform texture without detectable granules. These textural differences stem from the distinct manufacturing processes used for each type [27,35].

Biphasic fillers are typically produced with a minimal amount of cross-linking agents. The cross-linking conditions and duration are meticulously controlled to intertwine HA molecules into a cohesive and resilient gel mass that maintains structural integrity. This gel mass is then subdivided into smaller particles, preserving its granular nature, which contributes to the characteristic gritty texture of biphasic fillers. In contrast, monophasic fillers are formulated with a higher concentration of cross-linking agents, enhancing the bonding among HA molecules. Although the intermolecular bonds in monophasic fillers may be less robust than those in biphasic fillers, they encompass a broader surface area, resulting in a more extensively interconnected gel mass. This gel mass is subsequently ground into smaller particles, with the grinding process smoothing the particle surfaces and producing a range of particle sizes. This process results in the smooth, cohesive texture typical of monophasic fillers, which lack the granularity present in their biphasic counterparts [35].

Recent advancements in manufacturing techniques have led to the development of monophasic HA fillers with uniformly sized particles, exemplified by products such as Belotero (Merz Aesthetics, Raleigh, NC, USA) and HLLV (Sihler, Seoul, Republic of Korea). These innovations underscore the ongoing evolution of filler production methodologies.

A comprehensive understanding of these manufacturing differences is essential for distinguishing the physical properties of biphasic versus monophasic HA fillers. Each stage of the manufacturing process imparts unique characteristics to the fillers, thereby influencing their clinical performance and effectiveness. Continued research into these production methodologies is likely to yield deeper insights into the specific attributes that define each type of HA filler.

### 2.2. Hyaluronic Acid as a Material for Fillers

HA was first identified over eighty years ago in the vitreous body of bovine eyes, with its name derived from “hyaloid”, referring to the vitreous body, and “uronic acid” [1,2]. Subsequent extensive research has established HA as a ubiquitous component of the extracellular matrix in various human tissues, including the skin, synovial fluid of joints, vitreous humor of the eye, and cartilage scaffolding tissue. In an average adult weighing 70 kg, the total HA content is approximately 15 g, with about half of this amount, or 7–8 g, located within the dermis at concentrations ranging from 0.5 to 1 mg/g. HA is particularly notable for its exceptional moisture-binding capacity, with a single gram capable of retaining up to 6 L of water, thereby demonstrating its powerful hydrating properties while maintaining balanced moisture levels [2,3,4].

Approximately one-third of the total HA in the body, roughly 5 g, undergoes continuous synthesis and degradation each day, maintaining a steady quantitative equilibrium. HA synthesis occurs not only in dermal fibroblasts but also in epidermal keratinocytes. The half-life of HA is approximately one day, with complete turnover typically occurring within a week. Beyond its role in moisturization, HA is pivotal in protecting and stabilizing the skin by promoting cellular regeneration and providing structural support in conjunction with other components of the skin matrix. However, HA production diminishes with age, contributing to reduced skin moisture and elasticity [5].

Structurally, HA consists of repeating disaccharide units composed of N-acetyl-d-glucosamine and D-glucuronic acid, linked alternately via β-1.4 and β-1.3 glycosidic bonds, forming a glycosaminoglycan disaccharide. This repetitive disaccharide structure is consistent across both animal and bacterial sources. Consequently, HA used in filler production can be derived from either source; however, contemporary manufacturing practices predominantly utilize HA obtained through bacterial fermentation due to its consistency, purity, and reduced likelihood of provoking allergic reactions compared to animal-derived HA [6].

### 2.3. Hyaluronic Acid Filler Manufacturing Process

The transformation of HA into a filler product is a complex and multifaceted process involving several critical stages. Each stage is essential not only for understanding the properties of HA fillers but also for ensuring the production of safe, high-quality products. These stages significantly influence the defining characteristics of HA fillers and play a crucial role in determining the potential advantages and limitations of different formulations. A comprehensive understanding of these processes is imperative for both consumers and practitioners as it provides the foundation for establishing quality benchmarks and selecting HA fillers that are best suited to meet specific clinical needs [7].

### 2.4. HA Powder (Raw Material)

The raw material used in HA fillers, specifically HA sodium salt, is typically supplied in a powder form similar to flour. The quality of this raw material is paramount in the production of premium filler products as the use of high-grade HA significantly reduces the likelihood of adverse effects. HA naturally comprises long molecular chains capable of retaining substantial moisture; however, their large size limits their ability to penetrate the skin effectively. To enhance absorption through the skin’s stratum corneum, HA is processed into lower-molecular-weight forms, thereby improving its hydration and moisture retention capabilities. While low-molecular-weight HA excels in hydration, high-molecular-weight HA forms a protective barrier on the skin, preventing moisture loss, and medium-molecular-weight HA provides a balanced combination of moisture retention and barrier protection [8].

During purification, HA derived from microbial sources typically undergoes a reduction in molecular weight. Although microbial-derived HA generally ranges from 50 to 60 million Daltons, further purification is required for medical applications, particularly injectables, to produce HA powders with molecular weights between 50 and 3 million Daltons. The production of high-purity HA, essential for creating HA fillers, necessitates significant investment in production facilities, making it cost-prohibitive for some manufacturers. Typically, companies that produce medical-grade HA raw materials operate separately from those that manufacture HA fillers, although some pharmaceutical companies manage both processes depending on their operational needs [9].

The majority of HA filler manufacturers obtain their raw materials from suppliers listed in the Food and Drug Administration’s (FDA) Drug Master File, which includes producers from the European Union, the United States, Japan, and China. While some manufacturers have the technological capability to further process HA to enhance its purity, not all possess these advanced resources. Those without such capabilities often rely on the inherent high purity of their sourced materials to ensure product quality. The extent of processing and the purity level of the HA raw material play a critical role in determining the risk of inflammatory responses or hypersensitivity reactions in end users. Consequently, it is imperative for clinicians and consumers to verify the source and quality of the HA used in fillers to minimize the potential for adverse effects.

### 2.5. Dissolution and Cross-Linking of Hyaluronic Acid

To prepare an HA solution, the HA powder must first be dissolved in water, creating what is known as free HA. In its non-cross-linked form, free HA exhibits the physicochemical properties characteristic of a viscous fluid with low elasticity. Similar to natural HA, free HA is rapidly degraded by hyaluronidase upon injection, leading to its disappearance within a matter of days. To enhance its stability and transform it into a durable filler capable of maintaining volume, HA molecules require cross-linking. During the production of HA fillers, the addition of a cross-linking agent is essential for effectively binding the HA molecules, resulting in a cohesive and viscoelastic structure.

Achieving efficient cross-linking necessitates careful control of the temperature of the free HA during the process, as well as ensuring that the solution is stirred at the optimal speed and for the appropriate duration. This precise stirring process is critical as it facilitates the effective binding of the cross-linker to the HA molecules. Following the cross-linking reaction, the HA solution is cooled to solidify the newly formed bonds. This stage of dilution and cross-linking is pivotal in defining the rheological properties of HA fillers, making it a crucial step in the overall production process. Proper management of these parameters is essential to ensure the desired consistency, elasticity, and performance of the final HA filler product [10].

### 2.6. Types of Cross-Linking

Cross-linking is a fundamental process that enhances the stability of free HA, which naturally has a short half-life, by converting it into a more durable, three-dimensional structure. This process can be broadly categorized into two main methods: physical and chemical cross-linking. Physical cross-linking involves the formation of interconnections between HA chains, akin to tying knots in a rope. Various techniques are employed by different manufacturers to twist or coil these HA chains, preventing them from easily unraveling. These proprietary methods are often unique to each manufacturer, contributing to the distinct characteristics of their products [10].

In contrast, chemical cross-linking focuses on controlling the degree of cross-linking by adjusting the amount of cross-linker used, independent of the configuration or density of the HA chains. Common cross-linkers include 1,4-Butanediol Diglycidyl Ether (BDDE), Divinyl Sulfone (DVS), Bis-epoxides, and Polyethylene Glycol (PEG), selected for their established safety profiles and minimal toxicity. Currently, nearly all HA fillers utilize BDDE as the primary cross-linking agent [11].

As mentioned earlier, the molecular weight of sodium hyaluronate, the primary raw material used in injectable HA fillers, varies depending on the purification processes it undergoes. These processes result in molecular weights ranging from 500,000 to 3,000,000 Daltons. Medium-molecular-weight HA typically falls between 150,000 and 200,000 Daltons, while low-molecular-weight HA is generally around 100,000 Daltons or lower. High-molecular-weight HA, which exceeds 250,000 Daltons, is favored for its ability to enhance filler cohesiveness. However, it is more challenging to cross-link due to its sensitivity to temperature. Attempts to use even higher molecular weights in fillers have faced difficulties in achieving stable cross-linking, leading to product issues and, in some cases, market withdrawal. Consequently, high-molecular-weight HA generally remains within a relatively narrow range [12].

Low-molecular-weight HA, while advantageous for producing more condensed particles through strong cross-linking, requires rapid processing at high temperatures. This expedited process often results in the formation of “pendent types” of cross-links, where the cross-linking is incomplete, leading to potential drawbacks in the stability and uniformity of the filler properties. Medium-molecular-weight HA is considered the most stable for achieving prolonged cross-linking at room temperature, making it ideal for creating durable bonds. As a result, hyaluronic acid with molecular weights ranging from 1.5 to 2.5 million Daltons is commonly used in HA filler production [13]. Different manufacturers utilize raw hyaluronic acid with varying molecular weights. Although it was once believed that initial molecular weight differences in raw materials did not significantly impact the rheological properties of the final HA gel, modern manufacturing practices and product diversity suggest otherwise. The molecular weight of HA powder can influence the manufacturing process, making it advisable to verify the molecular weight of the hyaluronic acid used in a specific HA filler product [12].

**Figure 3:**
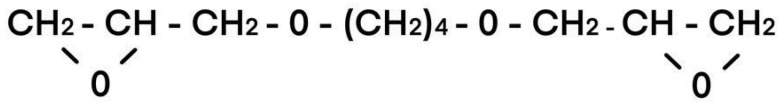
The structural formula of 1,4-Butanediol Diglycidyl Ether (BDDE).

The distinct physical properties of biphasic and monophasic fillers arise from differences in their cross-linking processes. Biphasic fillers are typically created using minimal cross-linkers (usually between 1% and 3%), with careful control of temperature, stirring speed, and reaction time. These conditions allow the HA chains to form long lines that naturally intertwine, creating a tangled, yarn-like structure that keeps the hyaluronic acid molecules cohesively bound. This method, known as physical cross-linking, focuses on adjusting the form of the cross-links rather than the number of molecules being linked, resulting in a firm gel mass that retains its shape under pressure (Figure 3) [14].

Monophasic fillers, on the other hand, are produced through a chemical cross-linking process where the degree of cross-linking is directly determined by the amount of cross-linker used, which influences the number of HA molecules involved. This method results in a gel mass that is generally softer and more pliable, often exhibiting a slightly sticky texture upon touch. Conversely, biphasic fillers utilize a minimal amount of BDDE as the cross-linker, leading to fewer HA molecules being chemically cross-linked. To compensate for this, the physical cross-linking between HA molecules must be intensified. This intensification requires an extended cross-linking time, as well as the careful application of controlled temperatures and slow, consistent stirring to ensure thorough mixing. When cross-linked over a prolonged period under mild conditions, the biphasic gel mass achieves a firm and elastic texture due to the tightly intertwined HA chains. In contrast, monophasic gel masses, characterized by extensive chemical bonding between molecules, exhibit a softer overall consistency, reflecting the distinct textural properties of this type of filler [14].

**Figure 4:**
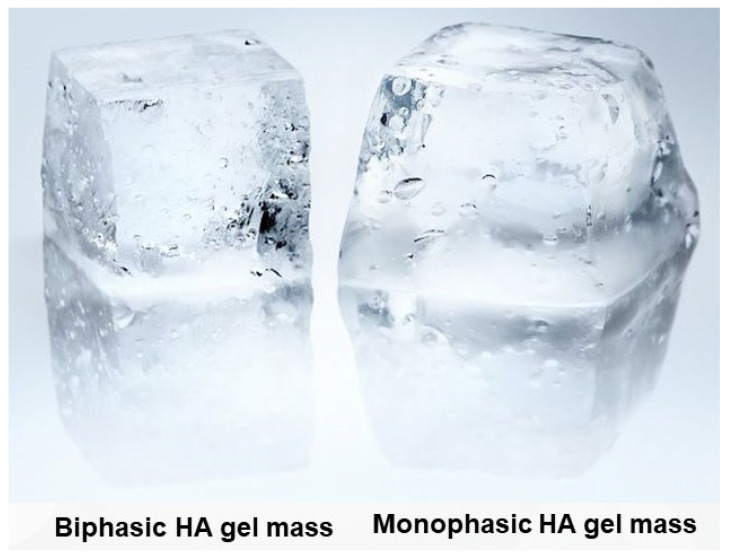
Comparison of external appearance of biphasic and monophasic HA gel mass.

Once the gel mass is formed, it is divided into smaller particles to create a product suitable for injection into the human body via a syringe. Typically, 1 mL of filler product consists of thousands to tens of thousands of particles, each retaining the properties of the original gel mass [15]. These particles collectively determine the overall characteristics of the product. As a result, biphasic gel masses, which are firmer and more elastic compared with monophasic gel masses, tend to feel grainy and rough after being processed into particles. In contrast, monophasic fillers possess a smoother and more uniform surface texture, with particles that are less perceptible to touch (Figure 4).

In the manufacturing of biphasic fillers, increasing the content of HA raw material can enhance the elasticity of the gel mass. By maintaining consistent conditions for the amount of BDDE and its interaction with free HA and simultaneously increasing the amount of HA powder, manufacturers can achieve a more concentrated and firmer gel mass. However, an excessive amount of HA raw material can result in a gel mass that is overly hard, thereby compromising the desirable viscoelastic properties of the filler. Therefore, manufacturers typically balance the amounts of BDDE and HA to produce a consistent and effective gel mass [14].

The viscoelastic properties of HA fillers play a crucial role in determining their performance and clinical applications. Viscoelasticity refers to a material’s ability to exhibit both elastic (solid-like) and viscous (fluid-like) behaviors under stress. In the context of HA fillers, these properties are influenced by factors such as the degree of cross-linking, molecular weight, and the specific manufacturing process. Biphasic fillers, which are minimally cross-linked, rely on natural molecular entanglements and typically display greater elasticity but lower cohesivity. This makes them suitable for applications requiring structural support and volume retention. In contrast, monophasic fillers undergo extensive cross-linking, resulting in higher cohesivity and a smoother, more malleable consistency, making them ideal for areas requiring seamless integration with surrounding tissues.

Understanding the rheological characteristics of HA fillers, such as the elastic modulus (G′), the viscous modulus (G″), and the complex modulus (G*), is essential for optimizing their use in clinical settings. These properties determine how the filler behaves under different types of stress, influencing factors like injection ease, tissue integration, and the longevity of the aesthetic results. For instance, a filler with a higher G′ value is more resistant to deformation and maintains its shape better under pressure, making it suitable for volumizing areas like the cheeks. On the other hand, fillers with a lower G′ are more appropriate for fine lines and wrinkles, where a softer, more fluid consistency is desirable. The balance between viscoelastic properties and safety is critical as it ensures that the fillers can achieve the desired aesthetic outcomes while minimizing the risk of complications.

The division of the gel mass into particles introduces variations in particle size, which in turn imparts distinct characteristics to each filler product. To maintain consistency, the concentrations of HA and BDDE are kept constant before the gel mass is divided into particles intended for use as fillers. The size of these particles significantly influences the clinical outcomes of the final product [14].

Larger particles, when maintaining the same volume, tend to create a more pronounced shape. It is crucial to understand that the firmness of each particle remains consistent, irrespective of its size, as all particles are derived from the same gel mass. The differences in the final product’s properties arise from how these particles aggregate. To illustrate, consider freezing water into a large block and then breaking it into smaller pieces. Larger chunks can form more substantial structures and melt more slowly compared with smaller ones, which do not create significant shapes and melt quickly. Similarly, in fillers, larger particles contribute to a more voluminous overall shape due to the greater space between them. In contrast, smaller particles gather more densely, resulting in a reduced overall volume [16]. Therefore, even though biphasic HA fillers are manufactured under consistent conditions, they may contain particles of varying sizes. However, the elastic properties of these fillers remain relatively uniform across different products. The slight differences in the observed elasticity are primarily due to the spatial variations created by the assembly of particles, which also affect the perceived volume and clinical appearance of the final product [17].

In contrast, the production of monophasic HA fillers involves adjusting the quantity of cross-linker while keeping the amount of HA constant. This variation in the cross-linker amount leads to the formation of gel masses with differing viscous consistencies. This process allows for the creation of fillers with a range of textures and consistencies, tailored to specific clinical needs and applications [14]. For instance, incorporating a higher concentration of cross-linker into the mixture results in a gel mass that is stickier and more cohesive, whereas using a lower concentration produces a softer, less adhesive gel mass. Consequently, when a monophasic gel mass is divided into particles for product formulation, it exhibits a smoother and more malleable consistency, lacking the grainy texture often associated with biphasic fillers and resembling a more viscous fluid.

Additionally, the particles formed from a monophasic gel mass do not maintain as firm or uniform a shape as those derived from biphasic fillers. As a result, the particle size in monophasic fillers has a diminished impact on the overall firmness of the product. Typically, monophasic fillers are produced by grinding the gel mass into particles of varying sizes, which reduces the significance of particle size in determining the filler’s properties. However, advancements in manufacturing techniques have enabled the production of monophasic fillers with controlled particle sizes, offering distinct advantages over those with irregular particle sizes. These developments will be further explored in subsequent discussions on the properties of HA fillers.

It is therefore important to recognize that when considering the elastic and viscous properties of HA fillers, it is inaccurate to attribute these characteristics solely to particle size and the amount of cross-linker used. The distinct cross-linking methods employed in the production of biphasic and monophasic fillers suggest that such a generalized view may be misleading. Users must be aware of the specific manufacturing processes behind the fillers they utilize as these processes significantly influence the consistency and performance differences between various types of fillers.

Before the gel mass is divided into particles, it undergoes additional purification processes, such as dialysis or washing. Depending on the manufacturer, the gel mass may be pre-cut into smaller sections prior to particle division, with some companies performing the cutting and particle division simultaneously.

### 2.7. Dialysis and Washing

**Figure 5:**
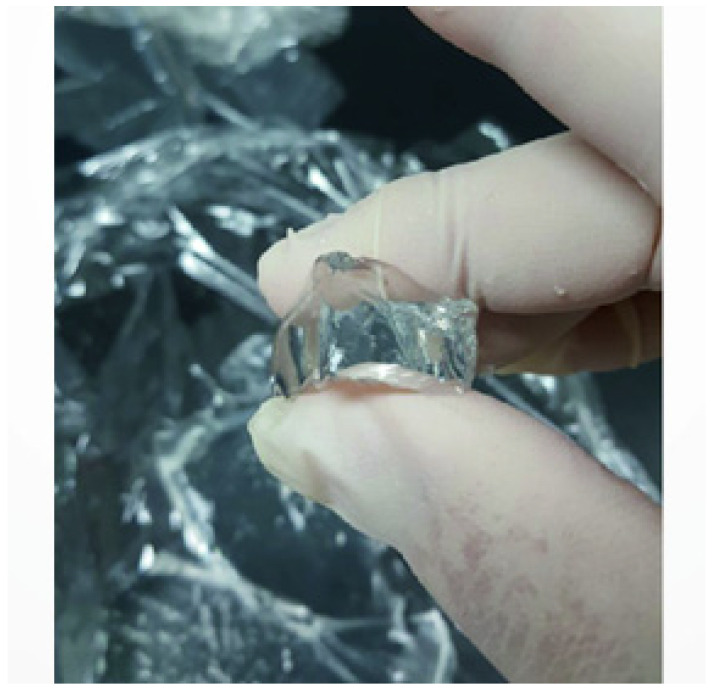
Hyaluronic acid gel mass segmented through a cutting process.

During the production of filler products, manufacturers employ dialysis or washing techniques to remove residual toxic substances, such as endotoxins and foreign materials, and to neutralize compounds following the cross-linking process. Maintaining a solution pH above 10 during cross-linking is crucial as it facilitates the reaction between 1,4-BDDE and free HA. The NaOH used to maintain this alkaline pH must be meticulously removed during the manufacturing process. This step is critical not only for adjusting the solution’s pH to appropriate levels but also for controlling osmotic pressure and ensuring the thorough elimination of any residual cross-linking agents, such as BDDE (Figure 5) [18].

The dialysis process in HA filler production typically involves two key stages: the first utilizes a sodium chloride solution, followed by a second stage employing a phosphate buffer solution. These steps are essential to ensure the safety and quality of the final product. To obtain approval from the FDA, HA fillers must meet stringent regulatory thresholds for endotoxins and residual levels of 1,4-BDDE. However, adhering to these minimum standards does not inherently guarantee complete safety. Consequently, most manufacturers strive to reduce endotoxins and residual BDDE to levels well below the regulatory limits.

Thorough dialysis or washing is vital for effectively eliminating toxins and foreign substances. Although this process is labor-intensive and time-consuming, often taking ten days or more, it is a critical step in producing high-quality HA fillers. When selecting HA filler products, it is imperative to verify that they have undergone sufficient dialysis or washing to remove potentially harmful residues, thereby ensuring their safety for clinical use.

### 2.8. Sieving or Grinding Process for Producing Gel Particles

**Figure 6:**
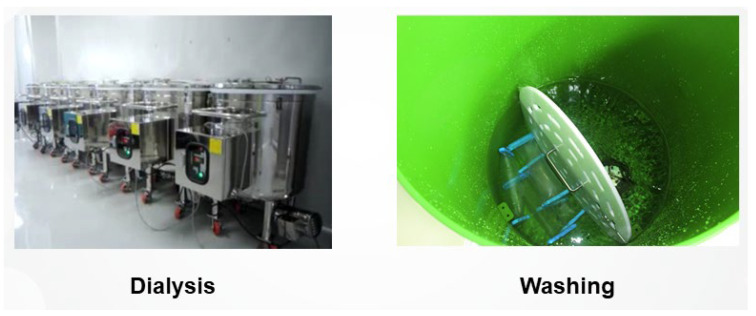
Dialysis and washing stages in hyaluronic acid filler production.

Following the dialysis or washing process, the gel mass must be subdivided into smaller particles to create the final filler product. The method of particle production differs depending on whether the gel mass is biphasic or monophasic. For biphasic gel masses, which are characterized by a firm and elastic texture, particles are typically formed through a sieving process. This involves passing the gel mass through screens or meshes of specific sizes to control the dimensions of the resulting particles. Larger particles are generally more suitable for contouring applications as they are more effective in maintaining and enhancing structural form (Figure 6).

Biphasic fillers typically consist of firm, granular particles. To facilitate injection through a syringe, these gel particles are sometimes mixed with a lubricant-like, non-cross-linked free HA. However, some products are composed entirely of small, compact granules without any free HA. When observed under a microscope, biphasic fillers that include free HA appear as solid particles suspended in a watery HA solution, creating the impression of two distinct materials combined. This visual distinction originally led to the term “biphasic”, which was used to describe the microscopic appearance of mixed substances, although it has since become widely adopted in popular terminology.

It is important to note that, whether in granular or mucilaginous form, all HA is chemically identical, rendering the term “biphasic” scientifically inaccurate. For products composed solely of granules without free HA, the term “biphasic” has been used to describe the texture imparted by the granules, but it is incorrect to use it to imply a mixture of two distinct substances. Additionally, contemporary monophasic fillers sometimes incorporate free HA to enhance injection smoothness, similar to biphasic fillers. Therefore, if HA fillers were to be strictly defined based on their composition, both biphasic and monophasic fillers—regardless of the presence of free HA solution—should technically be considered monophasic since they are composed entirely of HA molecules.

**Figure 7:**
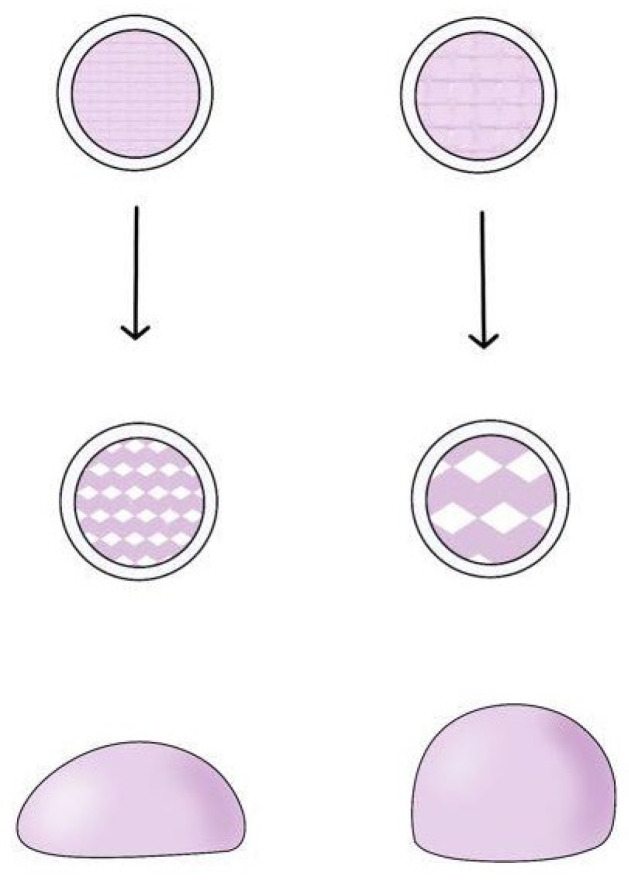
Sieving process used for particle sizing in biphasic hyaluronic acid filler production.

As a result, the terms “biphasic” and “monophasic” are now more accurately used to describe the shape of the particles formed during the manufacturing process rather than the type of substances they contain. Biphasic HA filler particles tend to have angular, multifaceted shapes, which is why the term “biphasic” is applied. In contrast, monophasic HA filler particles exhibit a uniform, rounded, single-surface appearance, leading to the term “monophasic”. To observe the shape of filler particles more accurately, one can dilute the filler product with water, agitate it to separate the particles, and then examine the individual particle shapes. This process clearly reveals the physical structural differences between biphasic and monophasic filler particles (Figure 7) [19].

**Figure 8:**
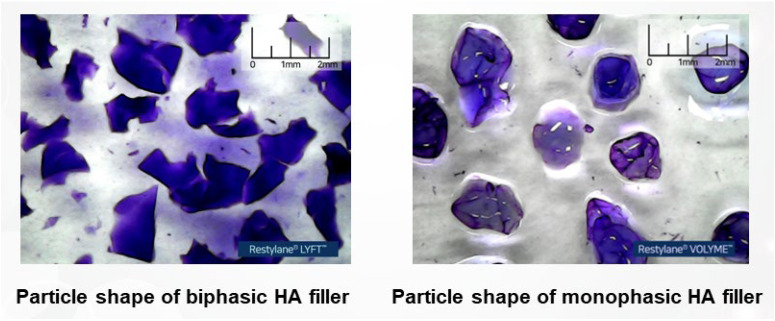
Comparison of particle shapes in biphasic and monophasic HA fillers.

Monophasic fillers are typically produced by grinding the gel mass into small, rounded particles that possess a smooth texture, akin to the consistency of pureed fruit (Figure 8). When HA fillers are stained and examined under a microscope, biphasic fillers generally display particles with jagged, coarse surfaces, resembling grains. In contrast, monophasic fillers appear as a single, cohesive, viscous mass without distinct particles, yet they maintain a smooth and uniform surface upon tactile examination.

Historically, monophasic fillers were often produced by grinding the gel mass into a homogenous, viscous consistency resembling thick fruit juice, typically without the inclusion of free hyaluronic acid (HA). To enhance viscosity, some monophasic fillers were formulated with a higher concentration of HA and increased cross-linking, resulting in a firmer texture that lacked the granular quality characteristic of biphasic fillers. In recent years, monophasic fillers have undergone more extensive cross-linking to improve their viscoelastic properties. To counteract the potential adverse effects on the injection experience caused by increased cross-linking, these fillers are often modified by blending in free HA, similar to the approach used in biphasic fillers, thereby achieving more advanced rheological properties in modern monophasic fillers.

**Figure 9:**
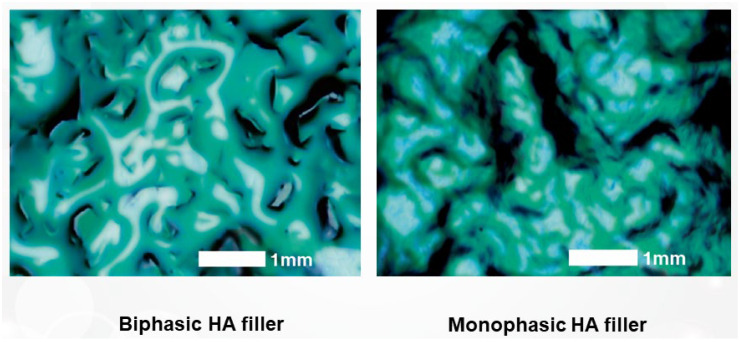
Microscopic comparison of biphasic and monophasic HA fillers.

**Figure 10:**
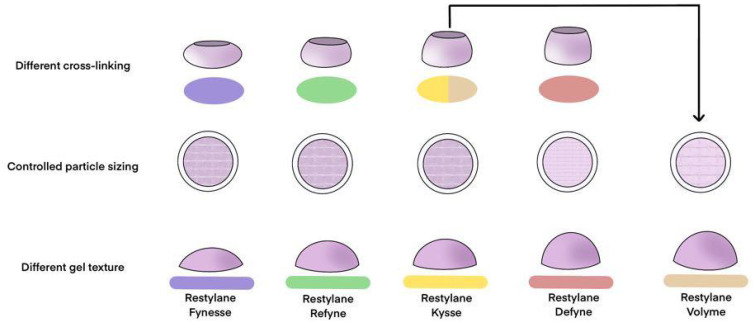
Sieving process used for particle sizing in Restylane OBT, a monophasic hyaluronic acid filler.

In the past, it was common to observe significant variation in particle size in monophasic fillers produced through grinding, rendering particle size less critical. However, contemporary manufacturing practices for monophasic fillers have evolved to achieve more uniform particle sizes by applying consistent pressure during the grinding process. Additionally, some monophasic fillers now employ screening techniques similar to those used for biphasic fillers to precisely control particle size (Figure 9 and Figure 10). 

Inadequate grinding of the gel mass can lead to significant variations in particle size, resulting in an uneven injection experience due to the presence of excessively large particles. Conversely, excessive grinding may cause thermal damage to the material, altering the rheological properties of the filler. Therefore, it is crucial to carefully control the grinding process to prevent these issues and maintain the desired characteristics of the filler.

As previously mentioned, the size of the particles in filler products produced through cutting techniques significantly influences the overall volumizing effect when these particles aggregate. The particle size also affects the extrusion force required during injection, which varies depending on the size of each particle. In areas where significant elasticity is not necessary, particle size can also impact the softness of the volume increase and the degree of cohesion between filler particles due to structural viscosity [16].

Many companies typically adjust their grinders to produce larger particles, which are better suited for enhancing volume. However, these larger particles often require the addition of free HA as a lubricant to facilitate smoother passage through needles or cannula tips during injection. Consequently, many modern monophasic fillers designed for high volumization, which involve extensive cross-linking to increase the filler’s consistency, are often mixed with free HA to alleviate stiffness and discomfort during use.

The inclusion of free HA not only serves a practical purpose but also influences the rheological properties of the product. Specifically, it affects the flexibility of the filler and its ability to return to its original shape after deformation, which is crucial for maintaining the desired aesthetic effect after application.

Recently, monophasic filler products with uniformly controlled particle sizes have been introduced, allowing for adjustments in material properties without the need to mix in free HA. Even for highly cross-linked fillers that traditionally required free HA for easier injection through needles or cannulas, it is now possible to administer filler particles using consistent force alone when particle sizes are uniform.

Monophasic fillers with uniform particle sizes reduce irregular spaces between particles when injected into the body, helping to achieve a more consistent and predictable shape. This uniformity minimizes the irregularities that can occur with mixed particle sizes. Additionally, as the filler degrades over time, the breakdown process of fillers with irregular particle sizes can lead to uneven changes in appearance, making it difficult to predict how the overall size and shape will evolve. In contrast, fillers with uniform particle sizes offer more predictable degradation patterns, maintaining a smoother appearance and allowing for more accurate forecasts of how the volume and shape of the filler will change over time.

### 2.9. Filling

**Figure 11:**
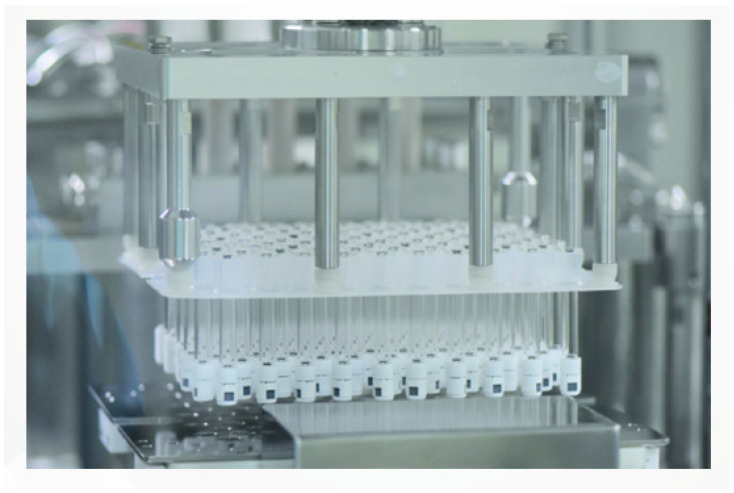
Filling process of hyaluronic acid fillers.

The gels are transferred into syringes and subsequently packaged as finished products. This filling process demands rigorous controls to ensure the prevention of contamination (Figure 11).

### 2.10. Sterilizing (Autoclave)

**Figure 12:**
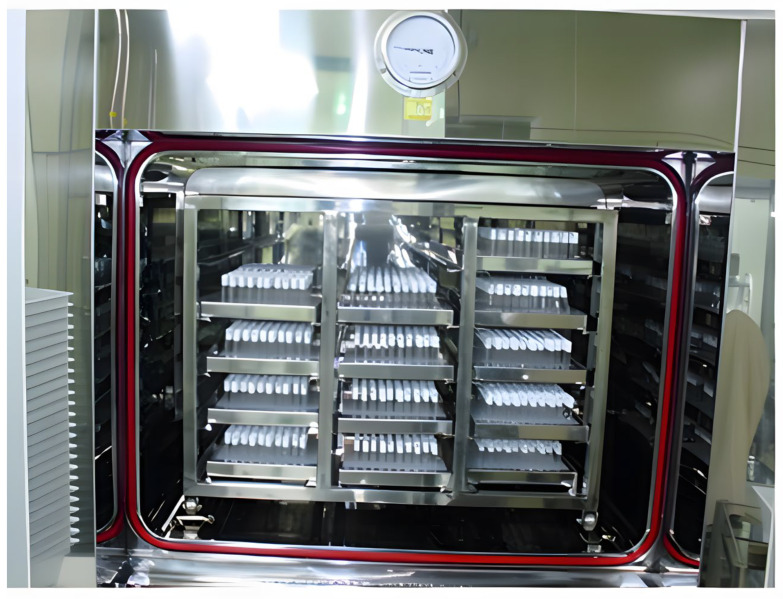
Sterilization process of hyaluronic acid fillers.

After the hyaluronic acid (HA) gel is loaded into syringes, the final HA filler products undergo a critical sterilization process. Sterilizing the filler after loading is essential because sterilizing the HA gel prior to filling could introduce subtle environmental variations during the sterilization procedure [36]. These variations have the potential to impact the cross-linking between HA molecules, thereby altering the gel’s viscoelastic properties and ultimately affecting the material characteristics of the finished product (Figure 12).

## 3. Non-Hyaluronic Acid Fillers

Apart from the early collagen fillers, which had shorter durations compared with HA fillers, most non-HA fillers are valued for their longer-lasting effects and their ability to stimulate more substantial collagen regeneration. Among non-HA fillers, those composed of biochemical substances that are not absorbed and remain in the body are classified as permanent fillers. However, due to their high potential for foreign body reactions and associated adverse effects, these permanent fillers are not commonly used in clinical practice [20,21]. Recently, there has been a growing interest in non-HA fillers not only for volumization but also for their regenerative properties, aimed at repairing damaged tissue. Notable examples include fillers derived from salmon sperm, such as polynucleotides (PNs) and polydeoxyribonucleotides (PDRNs), which are injected into the skin to support natural tissue repair processes [22,23,24,25].

### 3.1. Collagen Fillers

In the early development of fillers, initial biochemical substances caused significant side effects, ultimately leading to their prohibition [26]. However, with advances in modern medicine, fillers such as Zyderm and Zyplast—derived from bovine collagen—were developed and received approval from the FDA in the 1980s. Subsequently, collagen fillers sourced from other animals, such as porcine collagen (e.g., Permacol, Evolence), and human homologous collagen (e.g., Cymetra), were introduced [27]. Due to their animal origins, these collagen fillers necessitate a skin test prior to treatment to assess the risk of allergic reactions [28,29]. Initially, collagen fillers were less frequently used due to their shorter duration compared with HA fillers, which offered longer-lasting effects and thus became more prevalent. Recently, however, porcine-derived collagen fillers have been technically refined to last over a year [30]. Nevertheless, similar to earlier versions, no enzyme exists to dissolve collagen fillers once injected, necessitating careful consideration before use.

### 3.2. Calcium Fillers

Calcium fillers consist of two main components. The first is CaHA—Ca_10_(PO_4_)_6_(OH)_2_, a substance naturally found in the human body and used as a biocompatible material in medicine for decades. CaHA particles, sized between 20 to 45 microns, constitute about 30% of the total volume of the filler. Once injected into the skin, CaHA stimulates the surrounding tissues to promote collagen formation, and over time, it gradually degrades through metabolic processes into calcium and phosphate ions, which are then naturally eliminated from the body [26].

The second component, a gel carrier, composes the remaining 70% of the calcium filler’s volume and is composed of sodium carboxymethylcellulose, glycerin, and sterile water. The high molecular weight and viscoelastic properties of this mixture provide immediate volumizing effects post-injection and maintain the filler’s shape until the CaHA-induced collagen regeneration takes effect [31]. Clinically, compared with HA fillers, calcium fillers are known to facilitate more substantial collagen production as new collagen grows into the spaces created by the absorption of the gel carrier. The slow absorption of the gel carrier and significant collagen production contribute to a longer duration of effect compared with HA fillers. However, a notable disadvantage of calcium fillers is the absence of an enzyme that can dissolve them on demand, unlike HA fillers. Due to their excellent viscoelastic properties, calcium fillers are particularly useful in areas where HA filler treatments may have resulted in lower satisfaction regarding volume. Moreover, owing to the material’s characteristics, calcium fillers can also improve skin texture in addition to enhancing soft tissue volume [32].

### 3.3. Polycaprolactone Fillers

PCL fillers, widely used in thread lifting and scaffolding for nasal and breast surgeries, represent a significant advancement among polymers developed for medical applications. These fillers are composed of 30% smooth-surfaced, spherical PCL particles and 70% carboxymethylcellulose (CMC) gel carrier, resulting in fully absorbable components suitable for long-term medical applications [33,34].

Following injection, the CMC gel is gradually absorbed by macrophages over several weeks, while the PCL spheres, ranging from 25 to 50 µm in size, are not absorbed but become encapsulated by macrophages, thereby stimulating collagen proliferation. The newly formed collagen then occupies the spaces vacated by the absorbed gel carrier [35]. Clinicians using PCL fillers must be cognizant of the dynamic changes in volume that occur post-injection. Initially, swelling can create the illusion of increased volume, which diminishes as the swelling subsides. As the CMC gel is absorbed and replaced by collagen, the apparent volume may further decrease, only to increase in the subsequent weeks as collagen continues to proliferate. Patients may express concern that the filler seems to dissipate quickly between two to four weeks post-treatment; hence, caution is advised to avoid premature additional treatments, which may lead to overcorrection [37].

While long-lasting fillers like PCL are renowned for their durability and efficacy in collagen regeneration, they are also associated with a higher risk of delayed immune responses, potentially resulting in adverse effects. Generally, microspheres smaller than 15 µm are cleared via phagocytosis, whereas larger, irregularly surfaced microspheres may provoke inflammation and foreign body reactions, potentially leading to granuloma formation. A serious complication associated with long-lasting fillers is the development of foreign body granulomas, characterized by chronic inflammation and the formation of giant cells through macrophage fusion, which encapsulate the filler and form inflammatory masses [33,38,39]. Therefore, when selecting long-lasting fillers such as PCL, it is crucial to consider the potential for adverse effects. Given their propensity for extended collagen production, it is advisable to avoid overly superficial injections and to administer moderate amounts as no enzyme exists to dissolve these fillers, unlike HA fillers [33]. Some practitioners dilute calcium or PCL fillers with saline or lidocaine solutions to adjust the dosage and mitigate excessive collagen production. While dilution can facilitate smoother application, excessive dilution may compromise the homogeneity of the filler material and potentially damage the filler particles.

### 3.4. Poly-l-Lactic Acid Fillers

PLLA fillers, derived from polylactic acid, are widely recognized for their biocompatibility. Initially gaining prominence as a treatment for patients with significant facial lipoatrophy associated with AIDS, where other interventions such as fat grafting or alternative fillers were impractical, these fillers stimulate the body’s own collagen production to restore facial volume [40]. Unlike other fillers, PLLA comes in a powder form and requires reconstitution with sterile water, emphasizing its unique preparation requirements. After injection, the volumizing effects are not immediate; instead, facial contours gradually improve as collagen formation occurs over several weeks post-treatment [41]. Due to the gradual and somewhat unpredictable nature of collagen regeneration with PLLA, its primary use has shifted. While initially valued for restoring volume, it is now primarily utilized for its long-term effects on skin firmness and elasticity, enhancing structural integrity rather than providing immediate volume. This characteristic makes it particularly suitable for treatments aiming for subtle, natural-looking results and long-lasting improvement in skin texture [41,42]. PLLA fillers, including brands such as BLLV (Sihler, Seoul, Republic of Korea), are increasingly gaining popularity in Southeast Asia, reflecting a growing preference for their ability to achieve durable aesthetic enhancements.

### 3.5. Polymethyl Methacrylate Fillers

PMMA fillers, developed in 1992, consist of a blend of bovine collagen and PMMA, a synthetic polymer, in a 3:1 ratio. Initially employed as an implant material, PMMA was later adapted into a filler for aesthetic purposes, offering semi-permanent results [43]. In 2006, the FDA granted approval for ArteFill™, the commercialized form of these fillers [44]. However, due to its long-lasting efficacy, cautious use is recommended to mitigate potential adverse effects [45,46]. As a result, ArteFill™’s distribution is carefully managed by the manufacturer, limiting its broader adoption in clinical practice.

### 3.6. Polyacrylamide Gel Fillers

Similar to PMMA, Aquamid^®^ is a polyacrylamide hydrogel known for its biochemical stability and ability to yield semi-permanent results. Originally developed in Europe during the 1980s and initially marketed under the name Interfall, PAAG fillers gained increased recognition following the attainment of CE marking for Aquamid^®^ around 2000, facilitating its entry into global markets. However, the extensive utilization of PAAG in breast augmentation procedures in China resulted in multiple adverse effects, adversely affecting its reputation akin to liquid silicone and leading to a reduction in its widespread adoption [47,48,49].

## 4. Discussion

The primary clinical implications of this review on HA fillers and their manufacturing processes lie in improving filler selection and application to better meet specific patient needs, enhance safety, and optimize aesthetic outcomes. By providing an in-depth analysis of the distinctions between biphasic and monophasic fillers, the review equips clinicians with the knowledge necessary to choose the most appropriate product based on desired outcomes, such as volume enhancement or fine line smoothing. This understanding is crucial for minimizing adverse reactions, particularly with non-HA fillers that lack the reversibility offered by HA fillers. The ability to enzymatically dissolve HA fillers provides a significant safety advantage that clinicians should consider during treatment planning. Furthermore, this review offers valuable insights for making informed decisions in complex cases where the choice between HA and non-HA fillers could significantly impact patient outcomes. For instance, in cases requiring long-lasting effects or collagen stimulation, non-HA fillers may be preferable, but their irreversibility necessitates careful consideration of potential risks. The discussion on the viscoelastic properties of fillers further aids clinicians in predicting how different products will perform in areas of high facial movement, ensuring better aesthetic results that align with patient expectations.

The review of Wongprasert et al. [50] critically examined the current methods used to assess the properties of HA dermal fillers, which are popular for soft-tissue augmentation in cosmetic procedures. The authors highlight the lack of comprehensive data and standardized methods to compare different HA filler products, which complicates the process of selecting the most suitable filler for clinical use. The article critiques existing methods for characterizing HA fillers, such as rheology, swelling tests, enzymatic degradation assessments, and cohesion tests. These methods are essential for understanding how fillers will perform once injected into the body, particularly in terms of their ability to mimic natural tissue, resist deformation, and maintain their intended shape over time. The review underscores the importance of understanding the physicochemical properties of HA fillers, which vary significantly due to differences in cross-linking technologies, HA concentration, and manufacturing processes. It points out that while many studies focus on parameters like the elastic modulus (G′) and cohesion, these do not fully predict the long-term performance of fillers in vivo. The authors call for the development of standardized testing protocols that can better correlate the physicochemical properties of fillers with their clinical performance, concluding that a more thorough understanding of these properties, coupled with clinical experience, is crucial for optimizing the selection and use of HA dermal fillers in aesthetic medicine.

The manufacturing process and categorization of dermal fillers play a critical role in determining their safety, efficacy, and application in clinical settings. Among the most commonly used fillers, HA fillers stand out due to their biocompatibility and reversibility. HA fillers can be enzymatically dissolved, a significant advantage over non-HA fillers that lack a dissolving agent and require physical methods for removal [51,52]. Non-HA fillers, such as Ca-based, PMMA, PLLA, collagen, PAAG, and PCL fillers, are often chosen for their long-lasting effects and collagen-stimulating properties. However, they carry a higher risk of adverse reactions and complications if removal becomes necessary. This distinction underscores the importance of understanding the material composition and degradation mechanisms of different filler types [26,33,53,54,55].

Non-HA fillers differ from HA fillers primarily in their composition, longevity, and mechanism of action. Composed of materials like calcium hydroxylapatite (CaHA), polymethyl methacrylate (PMMA), poly-l-lactic acid (PLLA), polycaprolactone (PCL), and polyacrylamide gel (PAAG), non-HA fillers generally offer longer-lasting effects as they often stimulate collagen production, enhancing the skin structure over time. Unlike HA fillers, which can be easily reversed with hyaluronidase, non-HA fillers lack a dissolving agent, making them less flexible for adjustments and carrying a higher risk of complications. These fillers are typically used for deep tissue augmentation, facial contouring, and cases where durable, regenerative results are desired, but their permanent nature requires careful consideration due to the potential for more challenging adverse effects.

HA fillers, in particular, have significantly evolved in their manufacturing processes to enhance their physical properties and longevity. The transition from animal-derived sources to microbial fermentation has mitigated the risks of allergic reactions and pathogen transmission. Modern HA fillers undergo complex processes of cross-linking to prevent rapid degradation by natural enzymes, thereby extending their effectiveness [14]. The differentiation between biphasic and monophasic HA fillers lies in their cross-linking techniques and particle consistency. Biphasic fillers are characterized by a firmer, granular texture due to minimal cross-linking and careful control of temperature and stirring conditions, while monophasic fillers exhibit a smoother, cohesive gel mass achieved through extensive chemical cross-linking. Understanding these manufacturing details helps clinicians select the most appropriate filler based on the desired aesthetic outcomes and patient-specific needs, ensuring both immediate and long-term satisfaction.

Looking forward, the review highlights several emerging trends and future directions that are likely to shape the field of dermal fillers. One of the most promising advancements is the development of self-cross-linking hyaluronic acid (SC-HA) fillers. These innovative fillers differ from traditional HA fillers by utilizing a unique mechanism where the filler spontaneously cross-links with active oxygen present in the body after injection. This novel approach eliminates the need for chemical cross-linking agents, such as BDDE, which are commonly used in conventional fillers but may raise safety concerns. The self-cross-linking process, facilitated by a modified hyaluronic acid conjugate with gallol moieties, enhances both the biocompatibility and efficacy of the filler, improving its injectability and volumizing effects. SC-HA fillers represent a significant leap forward in filler technology, potentially offering safer and more effective options for tissue augmentation and wrinkle correction [56].

In addition to SC-HA fillers, the future of dermal fillers may also see the integration of bioactive substances that promote tissue regeneration and repair. Research is increasingly focusing on combining HA with growth factors, peptides, and other bioactive molecules that can enhance the filler’s ability to stimulate collagen production and improve skin quality over time [57]. This approach could lead to fillers that not only provide immediate aesthetic enhancements but also contribute to long-term skin health and rejuvenation. The integration of such bioactive components into filler formulations could open new avenues for personalized treatments, where fillers are tailored to meet the specific regenerative needs of each patient.

Another potential area of development is the use of nanotechnology in filler production. Nanotechnology offers the possibility of creating fillers with highly controlled particle sizes and distribution, which could result in more precise and predictable aesthetic outcomes. Nanoparticle-based fillers could also improve the delivery of active ingredients to targeted tissues, enhancing the efficacy and longevity of the treatment. Moreover, the incorporation of nanomaterials could allow for the development of multifunctional fillers that not only provide volume and contour but also deliver therapeutic agents for conditions such as hyperpigmentation or inflammation [58].

Furthermore, the trend toward minimally invasive procedures is likely to drive innovations in filler application techniques. Future advancements may include the development of delivery systems that minimize the discomfort and risks associated with injections, such as needle-free devices or advanced cannula technologies [59]. These innovations could improve the patient experience and expand the applicability of dermal fillers to a broader range of indications. Additionally, as the aesthetic industry becomes more attuned to sustainability and environmental considerations, there may be a shift toward the development of eco-friendly fillers. This could involve the use of biodegradable materials, sustainable sourcing of raw ingredients, and greener manufacturing processes. Such developments would not only reduce the environmental impact of aesthetic treatments but could also appeal to the growing segment of environmentally conscious consumers.

Last, there is potential for the development of smart fillers—formulations that can adapt to changes in the tissue environment. These smart fillers could respond to physiological changes, such as variations in pH or temperature, by altering their properties to maintain optimal performance [60]. For example, smart fillers could become more viscous in response to increased facial movement, ensuring that the filler remains in place and maintains its shape under dynamic conditions. The advent of smart fillers could revolutionize the field by providing adaptive, long-lasting solutions tailored to individual patient needs.

## 5. Conclusions

In conclusion, this review emphasizes the critical role of understanding the manufacturing processes and properties of HA fillers to optimize clinical outcomes. The distinctions between biphasic and monophasic fillers, their viscoelastic properties, and the limitations of current testing methods all contribute to the complex decision-making process in filler selection and application. As the field evolves, ongoing research and advancements in technology will likely lead to more refined and effective filler products, improved safety profiles, and enhanced aesthetic results. Clinicians should remain informed about these developments and consider both the current knowledge and future trends when planning and administering filler treatments to achieve the best possible outcomes for their patients.

## Data Availability

Not applicable.
